# Do people with ME/CFS and joint hypermobility represent a disease subgroup? An analysis using registry data

**DOI:** 10.3389/fneur.2024.1324879

**Published:** 2024-03-13

**Authors:** Kathleen Mudie, Allison Ramiller, Sadie Whittaker, Leslie E. Phillips

**Affiliations:** Solve M.E., Glendale, CA, United States

**Keywords:** joint hypermobility, myalgic encephalomyelitis, chronic fatigue syndrome, subgrouping, quality of life

## Abstract

**Background:**

Myalgic Encephalomyelitis/Chronic Fatigue Syndrome (ME/CFS) is a chronic, multifaceted disease that affects millions globally. Despite its significant impact, the disease's etiology remains poorly understood, and symptom heterogeneity poses challenges for diagnosis and treatment. Joint hypermobility, commonly seen in hypermobile Ehlers-Danlos Syndrome (hEDS), has been observed in ME/CFS patients but its prevalence and clinical significance within this population are not well-characterized.

**Objective:**

To compare the characteristics of ME/CFS patients with and without joint hypermobility (JH+ and JH-) as assessed using the Beighton scoring system, and to explore whether JH+ ME/CFS patients exhibit distinct disease characteristics, comorbidities, and health-related quality of life (HRQOL).

**Methods:**

The study used cross-sectional, self-reported data from 815 participants of the You + ME Registry. Participants were categorized as JH+ or JH- based on self–assessed Beighton scores and compared across demographics, comorbidities, family history, and symptoms. HRQOL was assessed using the Short Form-36 RAND survey and Karnofsky Performance Status.

**Results:**

15.5% (*N* = 126) of participants were classified as JH+. JH+ participants were more likely to be female, report Ehlers-Danlos Syndrome (EDS), Postural Orthostatic Tachycardia Syndrome (POTS), and a family history of EDS. They experienced worse HRQOL, particularly in physical functioning and pain, and a higher number of autonomic, neurocognitive, headache, gut, and musculoskeletal symptoms. Sensitivity analysis suggested that ME/CFS with concurrent JH+ and EDS was associated with more severe symptoms and greater functional impairment.

**Conclusion:**

ME/CFS patients with joint hypermobility, particularly those with EDS, demonstrate distinct clinical characteristics, including more severe symptomatology and reduced HRQOL. These findings highlight the need for comprehensive clinical assessments of ME/CFS patients with joint hypermobility. Understanding these relationships could aid in subgroup identification, improving diagnosis, and informing targeted therapeutic approaches. Further research is warranted to explore these associations and their implications for clinical practice.

## Introduction

Myalgic Encephalomyelitis/Chronic Fatigue Syndrome (ME/CFS) is a chronic, complex, systemic disease affecting anywhere from 1.5 to 3.4 million people in the United States (US), with an estimated annual economic cost of $36–$51 billion (1). ME/CFS can occur at any age and currently there is no correlation with race, or socioeconomic group; however, there is a clear sex bias with a female to male ratio of 3:1 (2). The cardinal symptom of ME/CFS is post-exertional malaise (PEM), a distinctive worsening of symptoms and functioning following physical, cognitive, emotional, sensory, or orthostatic stressors. Fatigue and neurocognitive manifestations are among the most reported and debilitating symptoms, but there exists substantial clinical heterogeneity in patients, who can experience a range of other symptoms, including orthostatic intolerance (OI), postural orthostatic tachycardia syndrome (POTS), brain fog, headaches, unrefreshing sleep, gastrointestinal issues, joint pain, and muscle pain. ME/CFS etiology is not established and biomarkers to distinguish the disease are not available, and so diagnosis occurs primarily based on clinical symptoms. However, inter-patient symptom heterogeneity and numerous associated comorbidities complicate diagnosis. Most clinicians lack the training and experience necessary to diagnose this complex disease and access to specialists is limited, leaving many patients undiagnosed or misdiagnosed (3, 4).

There are likely meaningful subgroups related to predisposing factors and disease characteristics that would allow researchers to disentangle risk factors and identify targeted and effective treatments. Genetic predisposition, disease trigger (e.g., infection), severity (house- or bed-bound), symptom clusters (e.g., dysautonomia symptoms), and comorbidities (e.g., hypermobility spectrum disorders) have been used to explore potential subgroups. However, diagnostic challenges and small, non-representative study sample sizes create persistent roadblocks to identifying homogenous subgroups (5). There is also notable selection bias in study participation, especially for in–person studies, which is more feasible for those less severely affected by ME/CFS.

Joint hypermobility, colloquially referred to as being “double-jointed”, describes one or more joints that stretch farther than normal. It is common, occurring in about 10–15% of the general population (6, 7). Like ME/CFS, females are affected about three times more often with joint hypermobility than males (8). A subset of people develops problematic, multi-systemic symptoms related to their hypermobility such as severe fatigue; problems with balance control; dizziness and fainting, especially when standing; gut, bowel, and bladder problems. These symptoms can indicate a more serious disorder that involves laxity (or looseness) of connective tissues, such as hypermobile Ehlers-Danlos Syndrome (hEDS). The co-occurrence of these conditions and overlapping symptomology with ME/CFS have been described (9, 10), but the prevalence and natural history of joint hypermobility and hEDS in the ME/CFS population is unknown. A 2021 study by Vogel et al. in a small observational cohort (*N* = 55) did not find evidence of any difference in clinical characteristics between hypermobile and non-hypermobile individuals with ME/CFS. However, the authors acknowledge that the detection of differences between groups might have been limited by small sample size.

The You + ME Registry is a secure, online real-world clinical data repository where people with ME/CFS, people with related diseases, and control volunteers enter information on their health that is then used for biomedical discovery (11). Compared to traditional, in-person studies, the Registry enables participation from people with diverse geography, backgrounds, and disease experiences (e.g., participation of severely ill patients who are house or bed-bound). The data collection includes validated questionnaires and patient-reported outcomes for researching associations between numerous characteristics and disease experiences. Disease subtype comparisons using data from registries have produced valuable insights, including clarification of clinical profiles and implication of targeted therapies (12, 13). Registries have been effectively used for other complex, heterogenous diseases, such as the Fox Insight study for Parkinson's disease (14), the IBD Partners Registry for irritable bowel disease (15), and ACCELERATE, an international registry for patients with Castleman disease (16).

The aim of this paper was to use Solve M.E.'s You + ME Registry to compare characteristics of ME/CFS participants with joint hypermobility (JH+) to those without (JH-), as evaluated using the Beighton scoring system. Widespread joint hypermobility is more often a congenital physiological condition present from birth, although it can be acquired (17). Assuming ME/CFS risk and clinical features are influenced by joint hypermobility and that joint hypermobility often temporally precedes onset of ME/CFS, we hypothesized that compared with participants without joint hypermobility, those with joint hypermobility have (i) an earlier age of ME/CFS onset; (ii) a gradual onset of ME/CFS and a lower likelihood of having their illness triggered by infection; (iii) presence of symptoms that relate to both ME/CFS and joint hypermobility; (iv) a greater prevalence of comorbidities, as well as a family history of related conditions; (v) worse health-related quality of life (HRQOL); and vi) more severe ME/CFS.

## Materials and methods

### Participants

Participants are from the You + ME Registry overseen by Solve M.E., a non-profit organization that works with international scientific, medical, pharmaceutical, and patient communities to lay the foundation for critical research into diagnostics, treatments, and cures for ME/CFS, Long COVID and other post-infection diseases. The protocol for You + ME Registry is described elsewhere (11). Briefly, participants are recruited through Solve M.E.'s social media channels (Facebook, Twitter, Instagram), via email to the Solve M.E. listserv, and promoted via webinars and conference presentations.

The registry is open to all individuals aged 18 years and older residing in the US and participants who self-identify as either a person with ME/CFS, a person with Long COVID, or as a control volunteer. After providing informed consent, participants complete a set of surveys, including the Symptoms Assessment developed by the UK ME/CFS Biobank (18) to ascertain ME/CFS case fulfillment according to the Fukuda Criteria (19, 20) or the Canadian Consensus Criteria (21). For this analysis, the Fukuda Criteria was modified to require PEM, the hallmark symptom of ME/CFS for a more homogenous group. ME/CFS participants who met either criteria and were not missing age or biological sex data were eligible for this analysis (*N* = 815).

### Measurement of joint hypermobility

**Figure 1:**
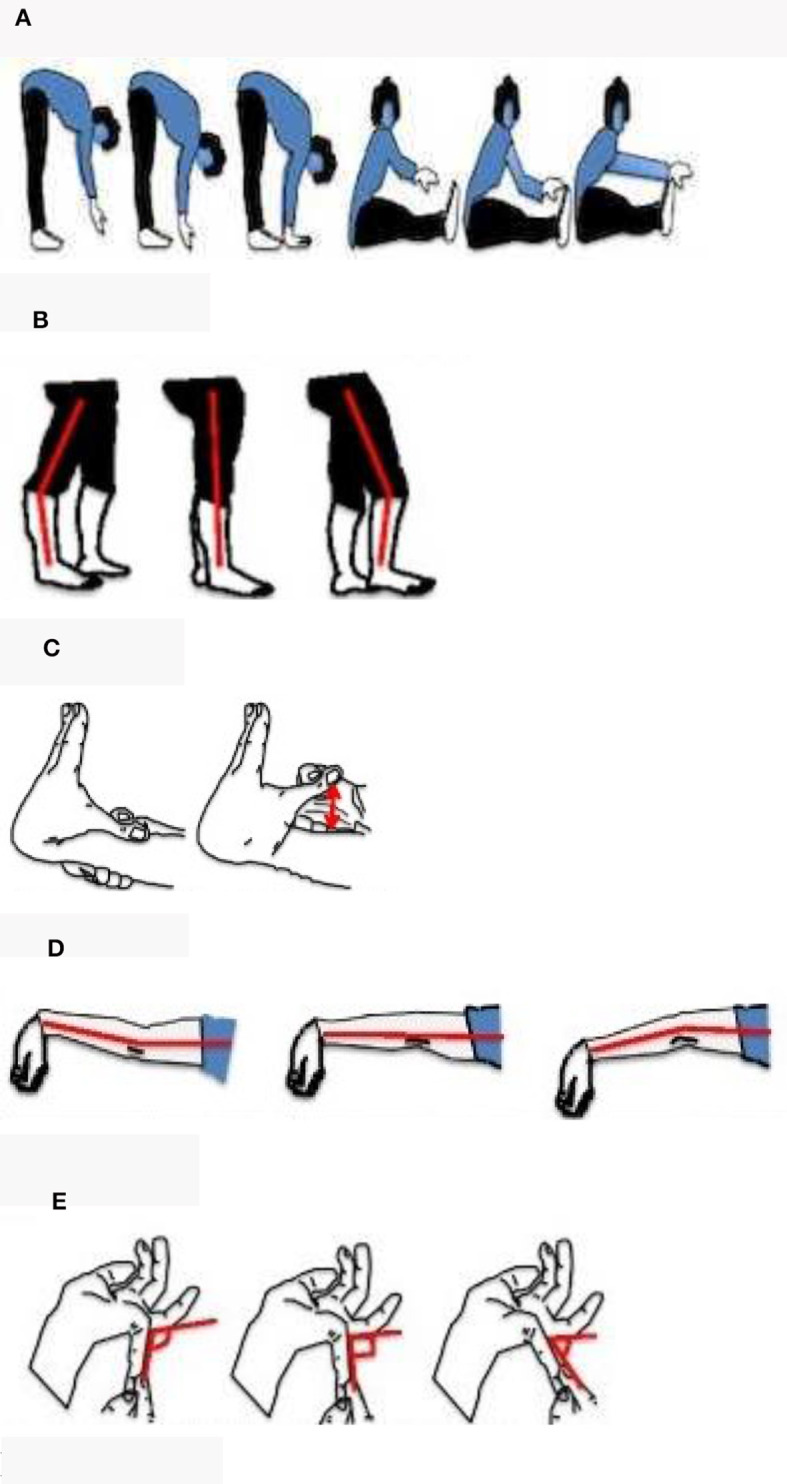
Self-reported line drawings of the Beighton score. Five sets of line drawings were created to depict the 9-point Beighton score criteria. Each instrument consisted of an explanatory question whereby participants were asked to select the line drawing which best represented their joints. **(A)** Trunk flexion: can't touch floor, fingertips touching floor, palms of hands on floor, can't touch toes, can touch toes, and can reach over toes. **(B–E)** Knee, elbow, and little finger extensions for each side of the body.

The Beighton Score was used to assess generalized joint hypermobility (presence of hypermobility in different joints throughout the body) (22). An individual's score is derived from a nine-point scoring system based on the performance of five maneuvers, four passive bilateral, and one active unilateral (Figures 1A–E) (22). The Beighton is used internationally to define joint hypermobility across all age groups and in diverse populations and has been shown to have good reliability and validity (23, 24). The Registry adopted a modified Beighton scoring system for self-reported joint hypermobility consisting of a series of electronic line drawings demonstrating the maneuvers (23).

Age-specific cut-offs were used to define joint hypermobility because joints become stiffer with age. Under 50 years old qualified as JH+ with a score of ≥5 and over 50 years old qualified as JH+ with a score of ≥4 (25). Thirty-nine participants were missing data for at least 1 question. Participants missing >2 responses were dropped from the analysis (*N* = 13). Participants missing up to two responses were excluded if their joint hypermobility status could not reasonably be inferred (*N* = 5). For example, if a participant was over 50 years of age with a score of 3 and was missing 1 question, the missing response was pivotal to determine their joint hypermobility status and they were dropped from analysis.

### Comorbidities

Participants were given an electronic form with open text fields to report their history of medical conditions. Generalized joint hypermobility is a diagnostic criterion for most EDS types and was included in our analysis, along with anxiety disorders, dysautonomia (e.g., POTS and hypotension), gastrointestinal disorders (e.g., IBS), ADD/ADHD, and Autism, as they are common comorbidities of joint hypermobility (17, 26, 27). Participants did not specify a particular subtype of EDS (e.g., hEDS). We also analyzed the total number of conditions reported.

### Clinical manifestations, course of disease, and risk factors

The Symptoms Assessment was used to capture the presence and severity (mild, moderate, severe, very severe) of symptoms related to ME/CFS, clustered into 12 groups: cold/flu, sensitivities, PEM, musculoskeletal, gastrointestinal, headaches, cognitive, sleep, autonomic, neuroendocrine, dermatological, and emotional. For the purpose of this paper, we focused on the presence of symptoms related to comorbidities common to joint hypermobility; for example, autonomic symptoms (dizziness/fainting, intolerance to standing, bladder problems, and palpitations), cognitive symptoms (brain fog, feeling lightheaded, loss of balance, and tingling/numbness in arms/legs), headache symptoms, gut symptoms, musculoskeletal symptoms (stiffness in the mornings, pain in two or more joints without swelling or redness, joint pains moving to different joints without swelling or redness, neck weakness, back weakness), and sleep symptoms. Dermatological symptoms are also present in some JH+ conditions, like EDS, but the questions in the Symptoms Assessment are not specific to those symptoms. Participants could choose an option for “I have NOT experienced any of these symptoms”; however, if a response was missing, it was assumed that the participant did not experience the symptom(s).

Additionally, participants were asked to provide demographic information (including age, biological sex, current pregnancy status, height, and weight), a detailed ME/CFS disease history, and diseases in their family history. BMI was calculated using self-reported height and weight. Participants with suspected anorexia (BMI < 17) or severe obesity (BMI > 40) were excluded because the former can cause extreme fatigue and is used as exclusion criteria for ME/CFS (20, 21) and the latter can interfere with range of motion (28, 29). From participants' ME/CFS disease history, we ascertained age at onset of ME/CFS symptoms (also used to calculate duration of ME/CFS), the timing of their disease onset (sudden ≤ 1 month vs. gradual > 1 month), and perceived trigger of their ME/CFS. We included data from participants' family disease history on diagnosed or undiagnosed ME/CFS and EDS because joint hypermobility can be both acquired (e.g., due to psychological distress, widespread inflammatory or degenerative diseases of the joints, past trauma/injury, athletic training) or inherited (17).

### Measurements of health-related quality of life

#### Short form 36-item health survey (SF-36)

The Short Form-36 (SF-36) developed by RAND is one of the most widely used generic measures of health-related quality of life (HRQOL) and has been shown to discriminate subjects with different severity levels of the same disease (30). The answers to the 36 questions form 8 subscales for physical functioning, role limitations due to physical problems, bodily pain, general health perceptions, vitality, social functioning, role limitations due to emotional problems and mental health (30, 31). Low scores indicate reduced HRQOL. The SF-36 is recognized as a reliable tool that has been validated across different populations and different chronic conditions and is used extensively in ME/CFS (19, 32, 33).

#### Karnofsky Performance Status scale

The Karnofsky Performance Status (KPS) Scale is an assessment of functional status that considers signs and symptoms of disease, activity level, ability, and assistance required (34). It has been shown to have good reliability and validity (35, 36). The scale is normally from 0 (dead) to 100 (normal, no complaints, no signs of disease) in units of 10. For the purpose of the Registry, the option of 0 (dead) was removed from the survey. A higher score indicates better functional ability and, therefore, less severe ME/CFS.

### Statistical analysis

ME/CFS participants with (JH+) and without (JH-) joint hypermobility were compared using Fisher's Exact test for categorical variables and either Wilcoxon rank-sum (for medians) or independent *t* tests (for means) for continuous variables. We considered a *p* < 0.05 to be significant.

### Sensitivity analysis

Clinical evidence suggests hypermobile EDS is more complex and severe than generalized joint hypermobility and other hypermobility spectrum disorders (37). To understand whether the characteristics under study in our ME/CFS cohort differed by hypermobility in the presence or absence of EDS, we performed a subgroup sensitivity analysis to compare: (1) JH+ with EDS to JH- without EDS and (2) JH+ without EDS to JH- without EDS. Thirty-one JH- with EDS were excluded from this analysis.

## Results

Of 3,592 ME/CFS participants in the You + ME Registry, 872 completed the Beighton and were eligible for this analysis (98% residing in the US), of which 15.4% (*N* = 134) qualified as JH+. Of note, 45 participants meeting inclusion criteria (15.6% with JH+) reported that their ME/CFS symptoms began after 13 January 2020 (the date of the index case of COVID in the US).

Table 1 displays characteristics of our study cohort overall and separated by whether they were JH+ or JH- according to Beighton. JH+ were significantly more likely to self-report EDS (29% vs. 3%, *p* < 0.001) and POTS (33% vs. 20%, *p* ≤ 0.001). JH+ participants had a higher prevalence of IBS, ADD/ADHD, Autism, and Hypotension, but the differences were not statistically significant. JH+ were also significantly more likely to report a family history of EDS (26% vs. 6%, *p* < 0.001) but not of ME/CFS.

**Table 1:** Characteristics of people with ME/CFS from the You + ME Registry overall and separated by whether they had joint hypermobility or not according to the Beighton Questionnaire; rows with cells < 5 participants were removed.

	**Total**	**Not hypermobile (JH-)**	**Hypermobile (JH+)**	***p-*value**
	***N*** = **872**	***N*** = **738 (84.6%)**	**134 (15.4%)**	
**Female sex assigned at birth** *N* (%)	752 (86.2%)	625 (84.7%)	127 (94.8%)	0.001^*^
**Current age** median (IQR)	49.00 (38.00-60.00)	50.00 (38.00-60.00)	46.00 (39.00-58.00)	0.089
**BMI (kg/m**^**2**^**)** median (IQR)	25.53 (22.30-30.33)	25.65 (22.38-30.41)	25.01 (21.74-30.13)	0.310
**Timing of onset: gradual vs. sudden** ***N*** **(%)**				0.240
Gradual > 1 month	427 (51.6%)	371 (52.6%)	56 (45.5%)	
Sudden ≤ 1 month	307 (37.1%)	253 (35.9%)	54 (43.9%)	
I don't know	94 (11.4%)	81 (11.5%)	13 (10.6%)	
**Infection as trigger** *N* (%)	533 (63.8%)	450 (63.4%)	83 (66.4%)	0.550
**Age at ME/CFS onset** median (IQR)	30.00 (17.00-42.00)	31.00 (17.00-43.00)	28.00 (16.00-38.00)	0.063
**Duration of disease** median (IQR)	14.00 (6.00-28.00)	14.00 (6.00-29.00)	16.00 (6.00-28.00)	0.870
**Comorbidities**				
**Ehlers-Danlos syndrome** *N* (%)	61 (7.1%)	23 (3.2%)	38 (28.8%)	< 0.001^*^
**Postural orthostatic tachycardia syndrome** *N* (%)	188 (21.8%)	144 (19.7%)	44 (33.3%)	< 0.001^*^
**Allergies** *N* (%)	406 (51.6%)	338 (50.4%)	68 (58.1%)	0.130
**Anxiety** *N* (%)	444 (51.5%)	382 (52.3%)	62 (47.0%)	0.300
**IBS** *N* (%)	691 (80.2%)	580 (79.5%)	111 (84.1%)	0.240
**ADD or ADHD** *N* (%)	107 (12.4%)	84 (11.5%)	23 (17.4%)	0.063
**Autism** *N* (%)	10 (1.2%)	7 (1.0%)	3 (2.3%)	0.190
**Hypotension** *N* (%)	12 (1.4%)	10 (1.4%)	2 (1.5%)	1.000
**Conditions count** mean (SD)	11.04 (11.36)	10.86 (11.42)	12.06 (11.06)	0.260
**Family history**				
**Ehlers-Danlos syndrome** *N* (%)	56 (8.7%)	33 (6.0%)	23 (25.6%)	< 0.001^*^
**ME/CFS** *N* (%)				0.630
No	579 (66.9%)	491 (67.1%)	88 (65.7%)	
Yes	176 (20.3%)	145 (19.8%)	31 (23.1%)	
I don't know	111 (12.8%)	96 (13.1%)	15 (11.2%)	
**QOL**				
**SF36 Physical functioning score** mean (SD)	34.61 (22.84)	35.30 (22.86)	30.65 (22.46)	0.036^*^
**SF36 Role limitations physical health score** mean (SD)	4.77 (13.31)	4.93 (13.60)	3.83 (11.51)	0.400
**SF36 Role limitations emotional problem score** mean (SD)	60.63 (43.06)	60.43 (43.16)	61.79 (42.61)	0.750
**SF36 Energy fatigue score** mean (SD)	9.27 (11.28)	9.44 (11.47)	8.31 (10.10)	0.300
**SF36 Emotional wellbeing score** mean (SD)	59.92 (20.57)	60.13 (20.70)	58.70 (19.81)	0.480
**SF36 Social functioning score** mean (SD)	26.74 (23.14)	27.27 (23.30)	23.69 (22.02)	0.110
**SF36 Pain score** mean (SD)	42.29 (23.51)	43.54 (23.54)	35.08 (22.04)	< 0.001^*^
**SF36 general health score** mean (SD)	25.06 (15.70)	25.37 (15.65)	23.27 (15.89)	0.170
**Karnofsky performance scale categories** ***N*** **(%)**				0.110
Mild/moderate impairment (60–90)	519 (63.1%)	447 (64.2%)	72 (56.7%)	
Severe/very severe impairment (10–50)	304 (36.9%)	249 (35.8%)	55 (43.3%)	
**Karnofsky score** median(IQR)	60.0 (40.0-70.0)	60.0 (40.0-70.0)	60.0 (40.0-70.0)	0.088
**Symptoms**				
**Autonomic symptom: dizziness/faintness while standing** *N* (%)	614 (70.4%)	512 (69.4%)	102 (76.1%)	0.120
**Autonomic symptom: intolerance to standing** *N* (%)	546 (62.6%)	446 (60.4%)	100 (74.6%)	0.002^*^
**Autonomic symptom: bladder problems** *N* (%)	483 (55.4%)	401 (54.3%)	82 (61.2%)	0.160
**Autonomic symptom: palpitations** *N* (%)	591 (67.8%)	488 (66.1%)	103 (76.9%)	0.016^*^
**Autonomic symptom: feeling lightheaded** *N* (%)	674 (77.3%)	562 (76.2%)	112 (83.6%)	0.072
**Autonomic symptom: any** *N* (%)	846 (97.0%)	715 (96.9%)	131 (97.8%)	0.780
**Autonomic symptom: count** mean(SD)	6.13 (3.06)	5.94 (3.00)	7.16 (3.15)	< 0.001^*^
**Cognitive symptom: brain fog**	800 (91.7%)	676 (91.6%)	124 (92.5%)	0.860
**Cognition symptom: loss of balance or inability to focus vision** *N* (%)	635 (72.8%)	526 (71.3%)	109 (81.3%)	0.015^*^
**Cognition symptom: tingling/numbness in arms/legs** *N* (%)	539 (61.8%)	442 (59.9%)	97 (72.4%)	0.007^*^
**Cognition symptom: any** *N* (%)	861 (98.7%)	729 (98.8%)	132 (98.5%)	0.680
**Cognitive symptom: count** mean (SD)	10.73 (3.39)	10.58 (3.38)	11.54 (3.35)	0.003^*^
**Headache symptom: migraines** *N* (%)	435 (49.9%)	357 (48.4%)	78 (58.2%)	0.039^*^
**Headache symptom: any** *N* (%)	733 (84.1%)	613 (83.1%)	120 (89.6%)	0.071
**Headach symptom: count** mean (SD)	1.54 (1.14)	1.50 (1.14)	1.77 (1.14)	0.012^*^
**Gut symptom: any** *N* (%)	769 (88.2%)	643 (87.1%)	126 (94.0%)	0.020^*^
**Gut symptom: count** mean (SD)	1.88 (1.03)	1.84 (1.03)	2.13 (0.96)	0.002^*^
**Muscle/joint symptom: stiffness in the mornings** *N* (%)	547 (68.6%)	466 (69.4%)	81 (64.3%)	0.310
**Muscle/joint symptom: pain in two or more joints without swelling or redness** *N* (%)	606 (69.5%)	518 (70.2%)	88 (65.7%)	0.020^*^
**Muscle/joint symptom: joint pains moving to different joints without redness or swelling** *N* (%)	595 (68.2%)	492 (66.7%)	103 (76.9%)	0.004^*^
**Muscle/joint symptom: neck weakness** *N* (%)	430 (49.3%)	348 (47.2%)	82 (61.2%)	0.038^*^
**Muscle/joint symptom: back weakness** *N* (%)	446 (51.1%)	366 (49.6%)	80 (59.7%)	0.060
**Muscle/joint symptom: any** *N* (%)	414 (47.5%)	340 (46.1%)	74 (55.2%)	1.000
**Muscle/joint symptom: count** mean (SD)	839 (96.2%)	710 (96.2%)	129 (96.3%)	0.002^*^
**Sleep symptom: any**	5.71 (2.52)	5.60 (2.53)	6.32 (2.40)	0.710
**Sleep symptom: count** mean (SD)	2.24 (0.92)	2.24 (0.92)	2.22 (0.93)	0.940

Some variables have missing data. Denominators were based on actual response count when determining the percentages. ^*^Statistically significant p-values.

Compared to JH-, JH+ had reduced HRQOL based on SF−36 Pain (35.1 vs. 43.5 mean, *p* < 0.001) and Physical Functioning (30.7 vs. 35.3 mean, *p* = 0.006) subscale scores. KPS scores suggest that the two groups had similar levels of functional impairment.

While JH+ had a higher prevalence of symptoms, only the following were statistically significant: the autonomic symptoms of intolerance to standing (*p* = 0.002) and palpitations (*p* = 0.016); neurocognitive symptoms of loss of balance or inability to focus vision (*p* = 0.015) and of tingling/numbness in arms and/or legs (*p* = 0.007); headache symptoms of migraines (*p* = 0.039); any gut symptom (*p* = 0.049); and musculoskeletal symptoms of pain in two or more joints without swelling or redness (*p* = 0.020), of joint pains moving to different joints without redness or swelling (*p* = 0.004), and of neck weakness (*p* = 0.038). When we looked at the number of symptoms reported by symptom cluster, JH+ reported a statistically significantly higher number of symptoms compared to JH-, except for sleep symptoms.

Other characteristics relevant to our hypothesis, including age of disease onset, suddenness of disease onset, and infectious trigger, were not found to be significantly different between groups.

Table 2 presents results from our sensitivity analysis comparing ME/CFS participants who were JH+ with EDS (*N* = 38) and JH- without EDS (*N* = 707). The JH+ with EDS group was younger at the time of data collection (*p* = 0.001). JH+ with EDS had a higher percentage of self-reported POTS (74% vs. 18%; *p* < 0.001); allergies (79% vs. 51%; *p* = 0.001), IBS (94% vs. 80%; *p* = 0.043), ADD/ADHD (26% vs. 12%; *p* = 0.018), and a higher number of conditions reported overall (mean 21 vs. 11; *p* < 0.001). HRQOL differences were evident in a significantly higher SF-36 Pain score (mean 29 vs. 44; *p* < 0.001) and more functional impairment as measured by KPS (median 40 vs. 60, *p* = 0.007) in the JH+ with EDS group. Individual symptoms present in significantly higher proportions in JH+ compared to JH- were also observed in JH+ with EDS compared to JH- without EDS, except for the musculoskeletal symptoms. The following symptoms were also more prevalent among JH+ with EDS: autonomic symptoms of dizziness/fainting while standing (*p* = 0.009), palpitations (p < 0.0001), and feeling lightheaded (*p* = 0.001); musculoskeletal symptom of neck weakness (*p* = 0.004); and headache symptom of migraine (p < 0.001). JH+ with EDS had significantly higher mean number of symptoms reported for all symptom clusters, except for sleep.

**Table 2:** Characteristics of people with ME/CFS from the You + ME Registry with joint hypermobility and EDS and those without joint hypermobility or EDS; rows with cells <5 participants were removed.

	**JH- without EDS**	**JH+ with EDS**	***p*-value**
	***N*** = **707 (94.9%)**	***N*** = **38 (5.1%)**	
**Female sex assigned at birth** *N* (%)	596 (84.3%)	35 (92.1%)	0.250
**Current age** median (IQR)	50.00 (38.00-61.00)	42.00 (33.00-49.00)	0.001^*^
**BMI (kg/m**^**2**^**)** median (IQR)	25.69 (22.40-30.41)	25.29 (21.97-29.84)	0.580
**Timing of onset: gradual vs. sudden** ***N*** **(%)**			0.180
Gradual > 1 month	357 (52.7%)	19 (51.4%)	
Sudden ≤ 1 month	244 (36.0%)	17 (45.9%)	
I don't know	77 (11.4%)	1 (2.7%)	
**Infection as trigger** *N* (%)	431 (63.2%)	24 (64.9%)	1.000
**Age at ME/CFS onset** median (IQR)	32.00 (17.00-43.00)	28.00 (15.00-32.00)	0.027^*^
**Duration of disease** median (IQR)	26.00 (15.00-39.00)	29.50 (23.50-41.00)	0.080
**Comorbidities**			
**Postural orthostatic tachycardia syndrome** *N* (%)	129 (18.2%)	28 (73.7%)	< 0.001^*^
**Allergies** *N* (%)	325 (50.6%)	27 (79.4%)	0.001^*^
**Anxiety** *N* (%)	368 (52.1%)	19 (50.0%)	0.870
**IBS** *N* (%)	561 (79.3%)	34 (89.5%)	0.150
**ADD or ADHD** *N* (%)	82 (11.6%)	10 (26.3%)	0.018^*^
**Autism** *N* (%)	6 (0.8%)	1 (2.6%)	0.310
**Hypotension** *N* (%)	9 (1.3%)	2 (5.3%)	0.100
**Conditions count** mean (SD)	10.64 (11.36)	20.97 (12.23)	< 0.001^*^
**Family history**			
**Ehlers-Danlos syndrome** *N* (%)	28 (5.2%)	15 (51.7%)	< 0.001^*^
**ME/CFS** ***N*** **(%)**			0.420
No	476 (67.8%)	22 (57.9%)	
Yes	136 (19.4%)	10 (26.3%)	
I don't know	90 (12.8%)	6 (15.8%)	
**HRQOL**			
**SF36 physical functioning score** mean (SD)	35.67 (22.88)	25.57 (22.15)	0.011^*^
**SF36 role limitations physical health score** mean (SD)	5.15 (13.86)	4.29 (9.56)	0.710
**SF36 role limitations emotional problem score** mean (SD)	60.29 (43.11)	73.33 (40.26)	0.080
**SF36 energy fatigue score** mean (SD)	9.50 (11.53)	7.14 (10.02)	0.230
**SF36 emotional wellbeing score** mean (SD)	60.25 (20.55)	61.41 (20.08)	0.750
**SF36 social functioning score** mean (SD)	27.39 (23.38)	22.50 (22.85)	0.230
**SF36 pain score** mean (SD)	43.85 (23.72)	28.50 (18.88)	< 0.001^*^
**SF36 general Health score** mean (SD)	25.61 (15.79)	20.86 (12.69)	0.080
**Karnofsky performance scale categories** ***N*** **(%)**			0.023
Mild/moderate Impairment (60-90)	215 (30.4%)	4 (10.5%)	
Severe/very Severe Impairment (10–50)	492 (69.6%)	34 (89.5%)	
**Karnofsky score** median(IQR)	60.00 (40.00-70.00)	40.00 (40.00-70.00)	0.007^*^
**Symptoms**			
**Autonomic symptom: dizziness/faintness while standing** *N* (%)	492 (69.6%)	34 (89.5%)	0.009^*^
**Autonomic symptom: intolerance to standing** *N* (%)	427 (60.4%)	34 (89.5%)	< 0.001^*^
**Autonomic symptom: bladder problems** *N* (%)	386 (54.6%)	22 (57.9%)	0.740
**Autonomic symptom: palpitations** *N* (%)	468 (66.2%)	36 (94.7%)	< 0.001^*^
**Autonomic symptom: feeling lightheaded** *N* (%)	541 (76.5%)	37 (97.4%)	0.001^*^
**Autonomic symptom: any** *N* (%)	692 (97.9%)	38 (100.0%)	1.00
**Autonomic symptom: count** mean (SD)	5.93 (2.95)	8.66 (2.64)	< 0.001
**Neurocognitive** symptom: brain fog	658 (93.1%)	37 (97.4%)	0.510
**Neurocognitive symptom: loss of balance or inability to focus vision** *N* (%)	507 (71.7%)	35 (92.1%)	0.004^*^
**Neurocognitive symptom: tingling/numbness in arms/legs** *N* (%)	426 (60.3%)	33 (86.8%)	< 0.001^*^
**Neurocognitive symptom: any** *N* (%)	706 (99.9%)	38 (100.0%)	1.00
**Neurocognitive symptom: count** mean (SD)	10.66 (3.20)	13.00 (2.84)	< 0.001^*^
**Headache symptom: migraines** *N* (%)	344 (48.7%)	30 (78.9%)	< 0.001^*^
**Headache symptom: any** *N* (%)	592 (83.7%)	37 (97.4%)	0.020^*^
**Headache symptom: count** mean (SD)	1.50 (1.12)	2.24 (1.15)	< 0.001^*^
**Gut symptom: any** *N* (%)	621 (87.8%)	38 (100.0%)	0.016^*^
**Gut symptom: Count** mean (SD)	1.84 (1.02)	2.39 (0.86)	0.002^*^
**Muscle/joint symptom: stiffness in the mornings** *N* (%)	502 (71.0%)	28 (73.7%)	0.850
**Muscle/joint symptom: pain in two or more joints without swelling or redness** *N* (%)	474 (67.0%)	32 (84.2%)	0.031^*^
**Muscle/joint symptom: joint pains moving to different joints without redness or swelling** *N* (%)	334 (47.2%)	27 (71.1%)	0.004^*^
**Muscle/joint symptom: neck weakness** *N* (%)	352 (49.8%)	28 (73.7%)	0.004^*^
**Muscle/joint symptom: back weakness** *n* (%)	328 (46.4%)	22 (57.9%)	0.180
**Muscle/joint symptom: any** *n* (%)	687 (97.2%)	38 (100.0%)	0.620
**Muscle/joint symptom: count** mean (SD)	5.63 (2.49)	7.00 (2.05)	< 0.001^*^
**Sleep symptom: any**	703 (99.4%)	37 (97.4%)	0.230
**Sleep symptom: count** mean (SD)	2.32 (0.91)	2.26 (0.92)	0.720

Some variables have missing data. Denominators were based on actual response count when determining the percentages. ^*^Statistically significant p-values.

Table 3 presents results from our sensitivity analysis comparing ME/CFS participants who were JH+ without EDS (*N* = 94) and JH- without EDS (*N* = 707). The SF-36 Pain score was significantly higher in JH+ without EDS (mean 44 vs. 38; *p* = 0.029) and the symptom of joint pains moving to different joints without redness or swelling occurred more frequently in this group compared to JH- without EDS (*p* = 0.048). JH+ without EDS had significantly higher mean number of symptoms in autonomic muscle/joint, and gut symptom clusters.

**Table 3:** Characteristics of people with ME/CFS from the You + ME Registry with joint hypermobility and without EDS and those without joint hypermobility or EDS; rows with cells <5 participants were removed.

	**JH- without EDS**	**JH+ without EDS**	***p*-value**
	***N*** = **707 (88.3%)**	***N*** = **94 (11.7%)**	
**Female sex assigned at birth** *N* (%)	596 (84.3%)	90 (95.7%)	0.002^*^
**Current age** median (IQR)	50.00 (38.00-61.00)	50.00 (41.00-59.00)	0.89
**BMI (kg/m**^**2**^**)** median (IQR)	25.69 (22.40-30.41)	24.75 (21.03-30.29)	0.620
**Timing of onset: gradual vs. sudden** ***N*** **(%)**			0.210
Gradual > 1 month	357 (52.7%)	36 (42.9%)	
Sudden ≤ 1 month	244 (36.0%)	36 (42.9%)	
I don't know	77 (11.4%)	12 (14.3%)	
**Infection as trigger** *N* (%)	431 (63.2%)	58 (67.4%)	0.480
**Age at ME/CFS onset** median (IQR)	32.00 (17.00-43.00)	29.50 (17.00-39.00)	0.380
**Duration of disease** median (IQR)	14.00 (6.00-29.00)	17.00 (6.00-28.00)	0.790
**Comorbidities**			
**Postural orthostatic tachycardia syndrome** *N* (%)	129 (18.2%)	16 (17.0%)	0.890
**Allergies** *N* (%)	325 (50.6%)	40 (48.8%)	0.081
**Anxiety** *N* (%)	368 (52.1%)	43 (45.7%)	0.270
**IBS** *N* (%)	561 (79.3%)	77 (81.9%)	0.680
**ADD or ADHD** *N* (%)	82 (11.6%)	13 (13.8%)	0.500
**Autism** *N* (%)	6 (0.8%)	2 (2.1%)	0.240
**Hypotension** *N* (%)	9 (1.3%)	0 (0.0%)	0.680
**Conditions count** mean(SD)	10.64 (11.36)	8.46 (8.21)	0.072
**Family history**			
**Ehlers-Danlos syndrome** *N* (%)	28 (5.2%)	8 (13.3%)	0.021^*^
**ME/CFS** ***N*** **(%)**			0.600
No	476 (67.8%)	64 (68.1%)	
Yes	136 (19.4%)	21 (22.3%)	
I don't know	90 (12.8%)	9 (9.6%)	
**HRQOL**			
**SF36 physical functioning score** mean (SD)	35.67 (22.88)	32.95 (22.32)	0.290
**SF36 role limitations physical health score** mean (SD)	5.15 (13.86)	3.69 (12.31)	0.350
**SF36 role limitations emotional problem score** mean (SD)	60.29 (43.11)	57.85 (42.67)	0.620
**SF36 energy fatigue score** mean (SD)	9.50 (11.53)	8.86 (10.16)	0.620
**SF36 emotional wellbeing score** mean (SD)	60.25 (20.55)	58.23 (19.09)	0.380
**SF36 social functioning score** mean (SD)	27.39 (23.38)	24.43 (21.77)	0.260
**SF36 pain score** mean (SD)	43.85 (23.72)	37.98 (22.69)	0.029^*^
**SF36 general Health score** mean (SD)	25.61 (15.79)	24.49 (16.85)	0.530
**Karnofsky performance scale categories** ***N*** **(%)**			0.560
Mild/moderate impairment (60–90)	434 (64.9%)	55 (61.8%)	
Severe/very severe Impairment (10–50)	235 (35.1%)	34 (38.2%)	
**Karnofsky Score** median(IQR)	60.00 (40.00-70.00)	60.00 (40.00-70.00)	0.630
**Symptoms**			
**Autonomic symptom: dizziness/faintness while standing** *N* (%)	492 (69.6%)	68 (72.3%)	0.630
**Autonomic symptom: intolerance to standing** *N* (%)	427 (60.4%)	66 (70.2%)	0.071
**Autonomic symptom: bladder problems** *N* (%)	386 (54.6%)	60 (63.8%)	0.098
**Autonomic symptom: palpitations** *N* (%)	468 (66.2%)	67 (71.3%)	0.350
**Autonomic symptom: feeling lightheaded** *N* (%)	541 (76.5%)	75 (79.8%)	0.520
**Autonomic symptom: any** *N* (%)	692 (97.9%)	93 (98.9%)	0.710
**Autonomic symptom: count** mean (SD)	5.93 (2.95)	6.70 (3.03)	0.018^*^
**Neurocognitive symptom: brain fog**	658 (93.1%)	87 (92.6%)	0.830
**Neurocognitive symptom: loss of balance or inability to focus vision** *N* (%)	507 (71.7%)	74 (78.7%)	0.18
**Neurocognitive symptom: tingling/numbness in arms/legs** *N* (%)	426 (60.3%)	64 (68.1%)	0.18
**Neurocognitive symptom: any** *N* (%)	706 (99.9%)	94 (100.0%)	1.00
**Neurocognitive symptom: count** mean (SD)	10.66 (3.20)	11.19 (3.00)	0.13
**Headache symptom: migraines** *N* (%)	344 (48.7%)	48 (51.1%)	0.66
**Headache symptom: any** *N* (%)	592 (83.7%)	83 (88.3%)	0.29
**Headache symptom: count** mean (SD)	1.50 (1.12)	1.62 (1.08)	0.34
**Gut symptom: any** *N* (%)	621 (87.8%)	88 (93.6%)	0.120
**Gut symptom: count** mean (SD)	1.84 (1.02)	2.07 (0.94)	0.039^*^
**Muscle/joint symptom: stiffness in the mornings** *N* (%)	502 (71.0%)	60 (63.8%)	0.150
**Muscle/joint symptom: pain in two or more joints without swelling or redness** *N* (%)	474 (67.0%)	71 (75.5%)	0.100
**Muscle/joint symptom: joint pains moving to different joints without redness or swelling** *N* (%)	334 (47.2%)	55 (58.5%)	0.048^*^
**Muscle/joint symptom: neck weakness** *N* (%)	352 (49.8%)	52 (55.3%)	0.330
**Muscle/joint symptom: back weakness** *N* (%)	328 (46.4%)	52 (55.3%)	0.120
**Muscle/joint symptom: any** *N* (%)	687 (97.2%)	91 (96.8%)	0.740
**Muscle/joint symptom: count** mean (SD)	5.63 (2.49)	6.18 (2.34)	0.044^*^
**Sleep symptom: any**	703 (99.4%)	94 (100.0%)	1.000
**Sleep symptom: count** mean (SD)	2.32 (0.91)	2.35 (0.95)	0.740

Some variables have missing data. Denominators were based on actual response count when determining the percentages. ^*^Statistically significant *p*-values.

## Discussion

The You + ME Registry includes data from over 2,000 people with ME/CFS. The size of the dataset provides a unique opportunity to pick apart the heterogeneity of ME/CFS and better understand disease subtypes.

Nearly 800 ME/CFS participants from the Registry cohort were included in this analysis to determine whether JH+ differed from JH- across a defined set of clinical characteristics. Joint hypermobility prevalence in the ME/CFS population is understudied. The proportion of ME/CFS JH+ in our sample was 15.5%, slightly lower than previous estimates of hypermobility prevalence in adult ME/CFS patient cohorts of 20% (38, 39) and much lower than Bragee et al., which reported 50% (40). Numerous factors might contribute to the observed prevalence differences, including the methodology used to classify patients as hypermobile and the characteristics of the patient populations themselves (e.g., clinic specialty focus on OI symptoms or more severe disease). There is also a possibility that the prevalence of joint hypermobility in the ME/CFS population is more accurately reflected in the Registry, which has a much larger sample size than previously reported studies. The lower prevalence in our patient sample ran counter to our expectation that the Registry might facilitate detection of joint hypermobility in patients with unrecognized disease or those who lack access to specialty care.

We found evidence that the JH+ group was more likely to have indications of hereditary hypermobility (e.g., a family history of EDS), a diagnosis of EDS, reduced HRQOL related to physical functioning and pain, and the presence of autonomic, cognitive, headache, gut, and musculoskeletal symptoms (without inflammation). We did not find any between-group differences for age of ME/CFS onset, timing of ME/CFS onset, infection as a precipitating event, or disease severity (as measured by KPS functional status). Participants with JH+ were more likely to be female compared to JH-, which is consistent with literature showing ME/CFS and JH+ (8, 26) are more common in females.

Our sensitivity analysis examining EDS in JH+ participants suggests that ME/CFS with JH+ might represent a heterogeneous group. When we looked only at JH+ with EDS compared to JH- without EDS, most differences observed in the larger group analysis were recapitulated with stronger statistical significance. Additionally, JH+ with EDS had a higher number of self-reported conditions, more functional impairment according to KPS, and a higher symptom burden. Most significant differences from the larger group analysis were not evident in our comparison of JH+ without EDS to JH- without EDS.

Headache, gastrointestinal manifestations, and dysautonomia are common among people with hypermobile EDS and often contribute to disability (41). Our sensitivity analysis showed an increasing prevalence of these symptoms in our ME/CFS sample along a gradient of JH- without EDS, JH+ without EDS, and JH+ with EDS. Chronic pain is one of the major symptoms presented by patients with hypermobile EDS (42); however, the SF-36 Bodily Pain score and prevalence of individual musculoskeletal symptoms in the JH+ with EDS group did not confirm pain as a differentiating symptom. JH+ without EDS was linked to increased pain levels, as well as an elevated burden of autonomic, musculoskeletal, and gastrointestinal symptoms compared to JH- without EDS. It is possible that some of these differences might be driven by undiagnosed cases of EDS in our data.

Overall, the sensitivity analysis showed the presence of comorbid EDS in our sample made differences between the JH+ and JH- groups more pronounced across many characteristics. The distinct patterns evident in our defined subgroups suggest that the presence of EDS is distinguishing clinically and that the differences observed in our comparison of JH+ and JH- were not attributable to joint hypermobility alone. However, incongruities in pain symptoms reinforce that complex factors and heterogeneity underlie these overlapping conditions and likely influence specific impairments and disease severity.

Although there is some evidence of an increased rate of JH+ reported in ME/CFS (9, 10, 43, 44) as well as an increased rate of ME/CFS in JH+ (8), previous studies by Castori et al. (8) and Vogel et al. (7) were unable to find any significant associations using much smaller sample sizes: 46 and 55, respectively. Our results partially corroborate Vogel et al. (7) who also failed to find an association between JH+ ME/CFS and earlier ME/CFS onset, more gradual ME/CFS onset, and infection as a trigger of their illness in a study of 55 children with ME/CFS. However, unlike Vogel et al., we did observe significant differences in symptomology and HRQOL indicators. There are many important differences between the Vogel et al. study and our study. Our study cohort was nearly 15 times larger, had an average age of was 39 years older (mean of 55 years in our study, compared with 16 years in Vogel et al.), and had a higher proportion of JH+ ME/CFS with a concomitant diagnosis of EDS.

Better understanding the relationship between ME/CFS, joint hypermobility, and EDS requires careful clinical assessment and consideration of underlying connective disorders in ME/CFS patients presenting with joint hypermobility. Characterizing these relationships may help identify subgroups of ME/CFS that respond to therapies targeting the precise biological mechanisms at play.

Our study includes some individuals who may have COVID-associated ME/CFS. While there is a lack of research on the association between JH+ and Long COVID, a preliminary study indicates that the presence of JH+ may be associated with a higher symptom burden in Long COVID patients, but the impact on quality of life was unclear (45). These observations in a Long COVID cohort and our study of ME/CFS suggest similar conclusions about the influence of joint hypermobility in these diseases. Commonalities between long COVID and ME/CFS have been previously reported (46), and the similarities in our findings underscore the importance of cross-disease comparative research. Further investigation into this relatively unexplored area of Long COVID research is needed.

### Strengths

We capitalized on a large dataset from the You + ME Registry to explore the theory of joint hypermobility as a subgroup of ME/CFS. Patient registries are invaluable resources for large-scale, real-world clinical data and bring new insights to the study of complex, heterogenous (14–16). The You + ME Registry data collection includes validated questionnaires frequently used in ME/CFS research that allow for replication of our methods by other researchers interested in this question. The low cost and reduced burden of administering the self-report Beighton via electronic questionnaire allowed us to achieve a much larger sample size with greater geographical diversity than is typical with an in-clinic assessment.

### Limitations

As with any research, there are limitations to the interpretation of our findings. The You + ME Registry is subject to selection bias, including socio-demographic and other differences between participants and non-participants, selective participant drop-off, and missing data. For example, patients with more severe disease and lower functional status might find the registration and data collection process too burdensome. Additionally, the Beighton was an optional questionnaire – available to those interested and able to complete it – and it is possible that response rates differed on characteristics relevant to our analysis. Currently, the Registry only integrates self-report data, which can produce measurement error due to participant recall, interpretation, or other factors (47).

Participants used a self-report version of Beighton, so our study lacks expert clinical assessment with goniometry to assess the degree of hyperextension. However, the self-report Beighton instrument showed strong agreement with expert clinical assessment in a pilot validation study (23). We lack other assessments possible with in-person studies, like arterial pulse wave velocity (PWV) to ascertain arterial stiffness and arterial elasticity, which would allow for (48) exploration of related questions regarding vascular connective tissue laxity.

The Beighton has not been established as a gold standard for assessing generalized joint hypermobility (19, 37, 41) and it is possible that study subjects were miscategorized as JH-. A low Beighton score does not necessarily rule out joint hypermobility because only select joints are examined (49), excluding common clinical sites of hypermobility, such as the cervical spine, shoulders, hips, and ankles. Furthermore, Beighton does not assess for other forms of connective tissue disease, like vascular EDS. Our study does not indicate Beighton can distinguish a clinical subgroup; however, it is a useful tool for assessing generalized hypermobility and the electronic self-report version could expand its utility for large-scale epidemiological studies in the ME/CFS population. Future studies should supplement the self-report Beighton with a targeted health history, including EDS diagnosis.

Our study only relied on current joint hypermobility status and did not collect historical data about hypermobile joints in childhood; therefore, it is possible that some participants below the Beighton cutoff in adulthood were hypermobile in childhood (40). Young age, female sex, and non-Caucasian ethnicity are associated with a higher prevalence of joint hypermobility (50, 51). Our analysis included age-specific Beighton score cutoffs but did not account for sex or ethnicity. Some studies propose age- and sex-specific Beighton score cutoffs (40, 52–54), but these explorations have been minimal and were not adopted for our study.

## Conclusion

Our analysis of a large, Registry-based population sheds light on the complex interplay between joint hypermobility, ME/CFS, and EDS. Our results showed distinctive clinical characteristics in ME/CFS with joint hypermobility, including a higher likelihood of hereditary hypermobility, reduced health-related quality of life (HRQOL) related to physical functioning and pain, and a range of autonomic, cognitive, headache, gut, and musculoskeletal symptoms. Sensitivity subgroup analysis underscored the importance of concurrent EDS. In this context, patients with both JH+ ME/CFS and EDS showed more severe symptoms, greater functional limitations, and an increased overall burden of symptoms compared to those with JH+ ME/CFS but without EDS. These findings emphasize the need for comprehensive clinical assessment and consideration of underlying connective tissue disorders in ME/CFS patients presenting with joint hypermobility. A comprehensive understanding of the clinical features, prognosis, and disease trajectory for these patients could guide cohort selection for research studies and facilitate the discovery of underlying disease mechanisms and targeted therapies. Further research is needed to understand the implications of joint hypermobility in ME/CFS for research, diagnosis, and clinical care.

## Data availability statement

The raw data supporting the conclusions of this article will be made available by the authors, without undue reservation.

## Ethics statement

The studies involving humans were approved by WCG IRB Connexus Review Solutions. The studies were conducted in accordance with the local legislation and institutional requirements. The participants provided their written informed consent to participate in this study.

## Author contributions

KM: Conceptualization, Data curation, Formal analysis, Investigation, Methodology, Project administration, Visualization, Writing—original draft, Writing—review & editing. AR: Conceptualization, Investigation, Methodology, Writing—original draft, Writing—review & editing. SW: Funding acquisition, Supervision, Writing—review & editing. LP: Supervision, Writing—review & editing.
